# Constructing a molecular interaction network for thyroid cancer via large-scale text mining of gene and pathway events

**DOI:** 10.1186/1752-0509-9-S6-S5

**Published:** 2015-12-09

**Authors:** Chengkun Wu, Jean-Marc Schwartz, Georg Brabant, Shao-Liang Peng, Goran Nenadic

**Affiliations:** 1 School of Computer Science, National University of Defense Technology, Changsha 410073, China; 2 Faculty of Life Sciences, University of Manchester, Manchester, M13 9PT, UK; 3 Department of Endocrinology, Christie Hospital, University of Manchester, Wilmslow Road, Manchester M20 4BX, UK; 4 Experimental and Clinical Endocrinology, Med Clinic I, University of Luebeck Ratzeburger Allee 160 D-23538, Lübeck, Germany; 5 Manchester Institute of Biotechnology, 131 Princess Street, Manchester M1 7DN, UK; 6 School of Computer Science, University of Manchester, Manchester M13 9PL, UK; 7 The Farr Institute of Health Informatics Research, Health e-Research Centre (HeRC), Manchester M13 9PL, UK

**Keywords:** Thyroid cancer, text mining, molecular interaction, pathway knowledge integration

## Abstract

**Background:**

Biomedical studies need assistance from automated tools and easily accessible data to address the problem of the rapidly accumulating literature. Text-mining tools and curated databases have been developed to address such needs and they can be applied to improve the understanding of molecular pathogenesis of complex diseases like thyroid cancer.

**Results:**

We have developed a system, PWTEES, which extracts pathway interactions from the literature utilizing an existing event extraction tool (TEES) and pathway named entity recognition (PathNER). We then applied the system on a thyroid cancer corpus and systematically extracted molecular interactions involving either genes or pathways. With the extracted information, we constructed a molecular interaction network taking genes and pathways as nodes. Using curated pathway information and network topological analyses, we highlight key genes and pathways involved in thyroid carcinogenesis.

**Conclusions:**

Mining events involving genes and pathways from the literature and integrating curated pathway knowledge can help improve the understanding of molecular interactions of complex diseases. The system developed for this study can be applied in studies other than thyroid cancer. The source code is freely available online at https://github.com/chengkun-wu/PWTEES.

## Introduction

Biomedical literature is a primary knowledge source for life science research, which facilitates the information and knowledge exchange through various biomedical studies. PubMed, the largest collection of biomedical literature, now contains over 24 million records [1]. In the past two decades, the annual increasing rate for the total citation count is around 4% [1].

This massive amount of available literature and its unstructured nature make it virtually impossible for researchers to keep track of all published results manually. Consequently, (semi-) automated methods and systems are needed to assist in the extraction of information and the reconstruction of knowledge. Text mining (TM) systems enable systematic collection of "scattered pieces" of information recorded in the biomedical literature [2,3]. This is particularly important for understanding biology at the systems level rather than "isolated parts of a cell or organism" [4]. Several TM systems have been developed, including named entity recognition (NER) and event extraction (EE) tools. NER tools can recognise mentions of key biological "named entities" in the literature, such as genes/proteins [5,6], diseases [7], species [8], pathways [9], etc. EE tools address the problem of extracting events, which represent specific relationships among entities. Typical molecular events include gene expression, gene regulation, binding, phosphorylation, transcription, protein catabolism and localization [10].

Curated databases constitute another important source of knowledge for biomedical studies. For instance, the 2014 Nucleic Acids Research online Molecular Biology Database Collection lists 1552 databases for molecular biology [11]. Specifically, a number of curated databases have been developed to represent the state-of-the-art knowledge of biological pathways, including the KEGG pathways [12], Reactome [13], WikiPathways [14] and Pathway Interaction Database (PID) [15]. Other databases like Pathway Commons [16] and ConsensusPathDB [17] incorporate and integrate information from multiple primary databases.

In this paper we present a methodology for constructing a comprehensive molecular interaction map of a disease by integrating the results of text mining with curated data for biological pathways. While current systems mainly focus on events involving genes and proteins only, our networks include both genes and pathways as nodes, where edges correspond to different interactions between them. Pathways in particular represent biological function organized temporally, and are therefore an important actor in interaction networks. We collect data about pathway interactions from the literature by expanding a state-of-the-art system for event extraction, the Turku Event Extraction System (TEES) [18]. The extended system, PWTEES (Pathway TEES), uses both genes and pathways as a type of entities involved in events.

To demonstrate the potential of combining gene and pathway interactions, we use thyroid cancer as a case study. Thyroid cancer is the most common endocrine malignancy [19] and its incidence has increased significantly over the past decades [20]. It is predicted that thyroid cancer will become the fourth most common cancer by 2030 [21]. We present a comprehensive molecular interaction network for thyroid cancer (576 nodes and 3136 edges), and discuss its properties using standard network metrics.

### Related work

Several efforts in mining complex and specific molecular events have been proposed [10,22,23]. In general, event extraction aims to locate the occurrence of an event, determine the type of event and assign its arguments. Systematically mined events can be used in various applications like semantic search engines, automatic database construction [24], and curation of biomedical knowledge [25,26].

A typical event extraction system will need to have multiple components: NER modules, parsing (to detect sentence grammatical structure and dependencies, in preparation for the relation detection), and relation extraction (determine the event type, participants of the event, etc.). Example event extraction systems include EventMine [27] and TEES [18,28]. TEES was reported as one of the best performing systems in the BioNLP'13 Shared Task challenge, with an accuracy of 50.75%. The system utilises results from a gene/protein NER (BANNER [6]) and a dependency parser to empower machine-learning based event detection. The event detection begins with trigger detection aiming at locating keywords that give hints about an event presence. Edge detection then determines the participants of detected event using a multi-class classifier that can determine argument types. In the final step, rules are employed to ensure that only one event node is associated with a trigger keyword.

Several datasets have been produced by applying event extraction tools to biomedical literature (e.g. BioContext [29], EVEX [22]). EVEX, for example, is an event database created by applying TEES on 21.9 million PubMed abstracts and 460,000 PubMed Central open access full-text articles. It contains 40 million bimolecular events and provides a web search interface for fast access to stored data [30].

While most of the current work focuses on gene/protein events, pathways have not yet been integrated into event extraction systems. Still, as an important concept, biological pathways have been frequently mentioned in the literature. Consider, for instance, the following sentence

**Notch pathway **is activated by **MAPK signalling **and influences papillary thyroid cancer proliferation. (PMID: 23544172)

This sentence expresses a relationship between two pathways (*Notch pathway *and *MAPK signalling*). However, existing EE tools would only detect events related to genes, and will ignore pathways. Even more, from the above sentence they might extract that the *Notch *gene is activated by *MAPK*, which is incorrect given that the interplay is between two pathways rather than two genes.

This example highlights the importance of introducing pathways in event extraction. In our previous work, we have described PathNER [9], a NER tool for pathway mention recognition. PathNER uses soft dictionary matching and rule-based methods, and has achieved an F1-score of 84% on a gold standard corpus. In this paper we integrate PathNER into TEES to support extraction of events that contain pathways.

In the area of cancer research, OncoSearch, for example, aims to detect gene expression changes in cancer-related MEDLINE abstracts [31]. It searches MEDLINE sentences for changes in gene expression levels and cancer status, and predicts gene roles (biomarker, oncogene, tumour suppressor gene, etc.). OncoSearch relies on BANNER for gene NER and TEES for gene expression identification.

As a case study to illustrate the potential of the proposed methods, we use thyroid cancer. In recent years, advances in the understanding of molecular pathogenesis of thyroid cancer have inspired novel biologically targeted therapies to further improve disease outcomes [32]. For instance, *vandetanib*, a tyrosine kinase inhibitor targeting the *RET*, vascular endothelial growth factor receptor (*VEGFR*), and epidermal growth factor receptor (*EGFR*), has been approved by FDA as a drug for medullary thyroid cancer. More potential targeted drugs are under investigation or being tested in clinical trials [33]. We have used text mining to construct a molecular profiling (related genes and pathways) of thyroid cancer, classified by commonly seen subtypes [34]. This has provided a systematic basis for the molecular understanding of thyroid cancer. However, details of the regulation patterns for those genes and pathways and involved interactions were not considered, and text mining methods have been highlighted as an important technology to address the problem of cancer gene and pathway prioritization [35].

## Methods

**Figure 1:**
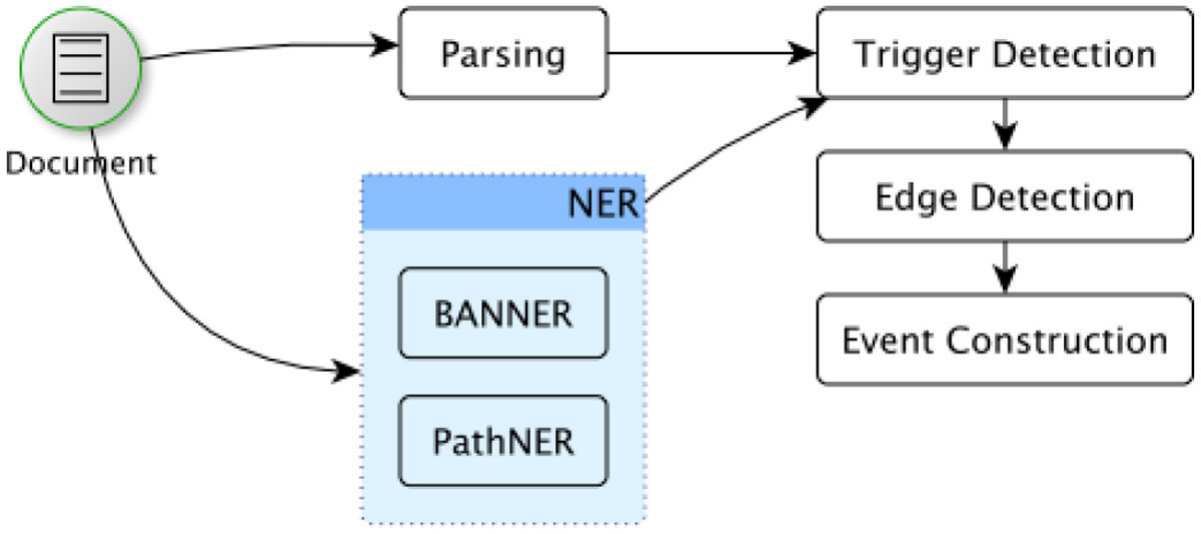
**Event extraction pipeline of PWTEES**.

We present here the steps needed to extract molecular events that involve genes/proteins and pathways, and use such data to construct an interaction network (see Figure 1). In particular, we describe PWTEES, an extension of TEES that includes pathways as named entities that are involved in molecular events.

### Recognizing gene and pathway mentions

We use BANNER for gene/protein name recognition and PathNER for pathways. The annotations from BANNER and PathNER are then post-processed to find overlaps. This is necessary because gene/protein names are frequently nested in pathway names [9]. In such cases, two overlapped mentions (one gene/protein mention and one pathway mention) are merged into one pathway mention by using the union of text boundaries.

### Entity normalization

Since BANNER does not provide normalization of recognised protein/gene names, we used GenNorm [36] to produce a mapping between mentions recognised by BANNER and Entrez Gene IDs. For pathway entities, genes contained in pathways were retrieved from the ConsensusPathDB database, 2013 edition [37]. As ConsensusPathDB integrates multiple pathway databases, there might be multiple different representations for the same pathway name. For instance, "Wnt signaling pathway" has multiple versions in KEGG pathways [12], WikiPathways [14], Pathway Interaction Database (PID) [15], and BioCarta (http://cgap.nci.nih.gov/Pathways/BioCarta_Pathways). In such cases, a union of all representations was performed.

### Event extraction

We used TEES to extract events. The original version of TEES only works with proteins/genes. We hypothesise that pathways appear in a similar context as genes/proteins when it comes to molecular events. A natural idea is thus to reuse the TEES machine learning based pipeline and its models. We did this by "disguising" pathway mentions recognised by PathNER as genes/proteins, with the same annotations to those produced by BANNER. In this extended version of TEES (PWTEES) we use the GE11 model that was used in the BioNLP'13 Shared Task challenge, which achieved an F1 score of 50.74% and was ranked second just after the 50.97% F1 score of the EVEX based method (based on TEES).

Not all types of molecular events can involve pathways. For instance, pathways cannot take part in gene expression, transcription, protein catabolism, phosphorylation, localisation and binding. Therefore, for pathway-involved events, we only consider *regulation, positive regulation *and *negative regulation*.

Events with gene/protein mentions that could not be normalized were discarded. Those mentions are likely *false positives *from BANNER. Similarly, events with pathway mentions that could not be mapped to ConsensusPathDB were removed. Those mentions are likely *false positives *from PathNER or they might not have been curated yet by the databases incorporated in ConsensusPathDB.

### Construction of interaction networks

Events usually involve arguments, including *themes *and *causes*. A *theme *is the entity being regulated in the event. A *cause *is the entity that regulates themes in the event. We are mostly interested in regulatory events that involve one theme and one cause, as well as binding events that involve multiple themes, as those events can provide explicit information about the interaction context for genes/pathways mentioned in literature. Note that directionality of events is ignored here.

Some events might take other events as theme or cause; in such cases, the nested events are expanded recursively to get all participating entities (either genes/proteins or pathway entities). For instance, in the sentence "...*HIPK2 *deficiency might be responsible for such paradoxical *Gal-3 *overexpression in WDTC." (PMID: 21698151), a positive regulation event is detected, where the theme and cause are both events (see Table 1).

**Table 1:** Example structure of a nested event.

Theme	T_Theme	*Gal-3*
	
	T_Cause	-
	
	T_Trigger	*Overexpression*
**Cause**	**C_Theme**	*HIPK2*
	
	**C_Cause**	-
	
	**C_Trigger**	*deficiency*
**Trigger**	*responsible*	

Example sentence "...*HIPK2 *deficiency might be responsible for such paradoxical *Gal-3 *overexpression in WDTC." (PMID: 21698151)

Events are represented as an interaction pair in the format of "<Cause, Theme>". Specifically for binding events, if more than two themes are involved, e.g., <Theme1, Theme2, Theme3>, then resulting interactions will contain multiple pairs: <Theme1, Theme2>, <Theme1, Theme3>, and <Theme2, Theme3>.

We then construct a molecular interaction network, where the nodes are genes and pathways, and the edges are added in the following way:

**1) Text-mined results**: all interactions detected by PWTEES are added to the edge set.

**2) Curated data**: we integrate curated molecular knowledge about pathways into the network as follows: if a gene node A is contained in a pathway C (as specified by ConsensusPathDB), then a new edge <A, C> is added to the edge set.

### Data and large-scale processing

The corpus used in this study contains 38,572 abstracts from MEDLINE, as described in [34]. It was constructed by the PubMed query "*(((thyroid neoplasms[majr] AND human[mh] AND english[la]) OR thyroid[ti]) AND (cancer OR carcinoma OR malignant OR malignancy))*", as suggested by the National Cancer Institute (http://www.cancer.gov/types/thyroid). The results were limited to human studies in English.

**Figure 2:**
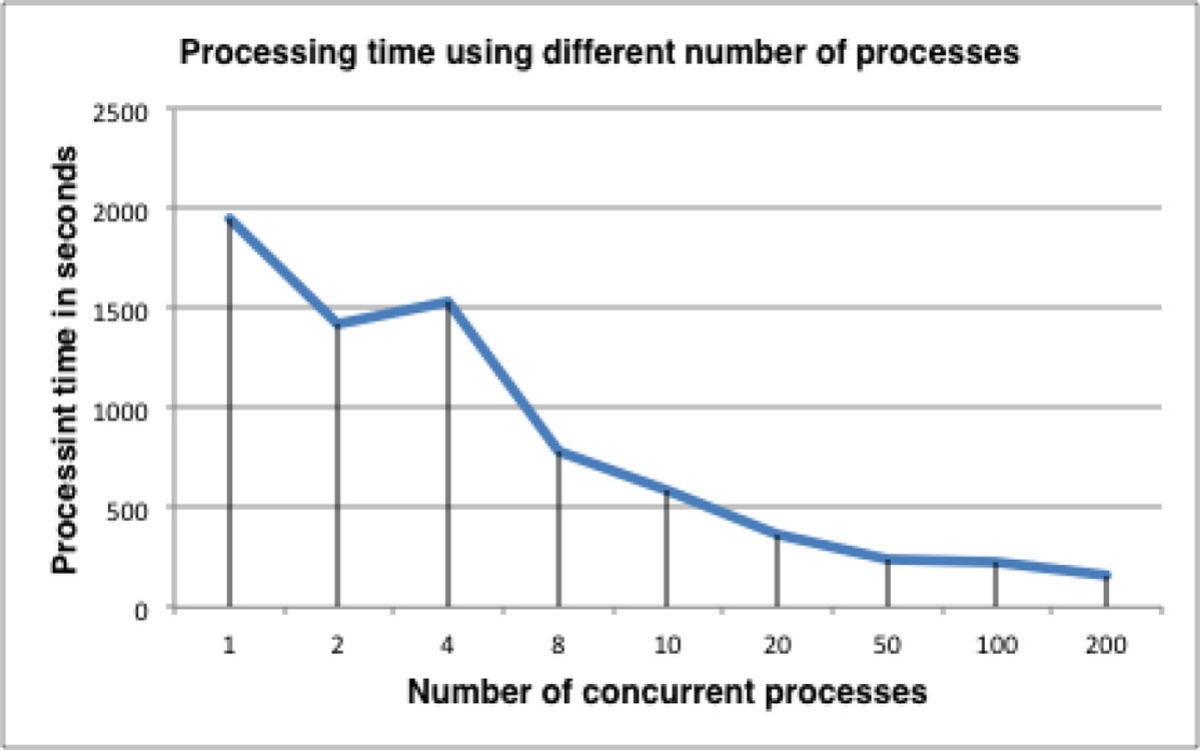
**Effects of parallel processing on processing time**.

It is a computationally time consuming process to perform event extraction on such a large collection of documents. To improve efficiency, we employed parallel processing. We implemented the whole pipeline of PWTEES on the world's fastest supercomputer Tianhe-2 built by the National University of Defense Technology (http://top500.org/featured/top-systems/tianhe-2-milkyway-2-national-university-of-defense/). We carried out tests with 1,000 randomly selected abstracts from MEDLINE: the processing time was less than 3 minutes using 200 processes (initialization of the pipeline takes about two minutes for each process), as depicted in Figure 2. Using a larger pool of compute nodes (6,000 concurrent processes), we were able to finish the processing of the whole thyroid cancer corpus within 3 minutes.

## Results and discussion

### Evaluation of PWTEES

For molecular events that do not involve pathways, PWTEES is equivalent to TEES, which has already been thoroughly evaluated [38]. Here we therefore focus on evaluating the performance of PWTEES on *pathway events *specifically. To note, in this study, pathway events are defined recursively. An event that takes a pathway as a theme or cause is a pathway event; an event that takes another pathway event as its theme and cause is also a pathway event. A pathway event is considered correct only if the event and the arguments are both correct.

We evaluated PWTEES in two ways: firstly, we randomly sampled 100 reported pathway events (named as ***P_TEST***) to evaluate the precision of PWTEES on pathway event detection; secondly, to estimate recall, we constructed a set of 100 <pathway, gene/protein> pairs (named as ***PR_TEST***), which were randomly sampled from all possible <pathway, gene/protein> pairs satisfying the following two conditions: (1) the entities in one pair appear in the same document; (2) their distance is smaller than 100 characters (an empirically selected threshold). The PR_TEST set is used to estimate the recall of PWTEES (named as *pseudo-recall *herein), given by:

$$pr=\frac{TP}{TP+FN}$$

where TP is the number of true positives by PWTEES in the PR_TEST; FN is the number of false negatives by PWTEES in the PR_TEST. We could not calculate the genuine recall, which would require random sampling from the whole possible space of events. This is mainly because the density of pathway events is much lower than that of gene/protein events, which could require a large number of samples in order to get sufficient true pathway events to reflect the true recall of PWTEES.

The details of the performance evaluation are listed in Table 2. The precision calculated on the P_TEST is 72% and the pseudo-recall calculated from PR_TEST is 50%. This gives a rough estimated F1-score of 59%.

**Table 2:** Performance evaluation of PWTEES.

Dataset	TP	FP	TN	FN	P	PR
P_TEST	72	28	-	-	72%	-

PR_TEST	10	3	77	10	-	50%

TP: true positives; FP: false positives, TN: true negatives; FN: false negatives; P: precision; PR: pseudo-recall.

We analysed several typical types of error in the pathway events reported by PWTEES, as listed in Table 3. Typical errors include wrong assignment of arguments, wrong event types, and missed arguments. False negatives of the gene NER component can cause a miss of an important argument, as demonstrated in example #3 in Table 3 in which BANNER could not detect *G691S *(*RET Exon 11 *polymorphism). Some of the errors were also present in TEES. For instance, in example #4 in Table 3, PWTEES reports a pathway event with the theme to be a simple phosphorylation event. For this example, the theme of the phosphorylation should be nucleoproteins while the cause should be *MAPK*.

**Table 3:** Examples of pathway event errors.

PMID	Sentence	PWTEES	Comment
8875985	...directed the expression of either the A2a adenosine receptor that constitutively activates the cAMP pathway, or the E7 protein	**T**: *cAMP cascade* **C**: *E7 *protein **ET**: Positive regulation	The cause should be A2a adenosine receptor. Wrong assignment of argument.

23261982	Dkk-1 inhibited the survival and migration of human PTC cells by regulating Wnt/β-catenin signaling and E-cadherin expression.	**T**: *expression of Wnt/beta-catenin signaling and E-cadherin* **C**: *Wnt/beta-catenin signaling pathway* **ET**: *Regulation*	Cause should be Dkk-1 and event type is inhibition. Wrong assignment of argument and event type.

21690267	...the same patient allele carries both K666E and G691S variants, the latter known to increase downstream RET signaling,	**T**: *RET downstream signaling* **C**: **ET**: *Positive regulation*	Cause should be *G691S variants*. Missed argument

15059947	...the MAPK (ERK1/2) signaling pathway causes serine phosphorylation by MAPK of several nucleoproteins	**T**:{T:MAPK; Site: serine; ET: Phosphorylation} **C**: MAPK (ERK1/2) signaling pathway **ET**: Positive Regulation	In the nested theme event, MAPK should be the cause, not the theme.

16940797	ZD 6474 has shown promising activity in preclinical models against RET kinase, and its contemporary inhibition of vascular endothelial growth factor and epidermal growth factor pathways	**T: **VEGF and EGF pathways **C:** **ET**: Negative Regulation	Cause should be ZD6474 (drug).

T - Theme, C - Cause, ET - Event Type

We also noticed several PathNER errors. In example #2 in Table 3, *Dkk-1 *inhibits "the survival and migration of human PTC cells by regulating Wnt/β-catenin signaling and E-cadherin expression". However, PathNER reports "Wnt/β-catenin signaling" and "Wnt/β-catenin signaling and E-cadherin expression" at the same time, which was not correct as pathway mentions should not overlap. In the same example, the event type was incorrect, which could be possibly caused by the complex structure of the sentence and the presence of multiple event trigger keywords ("inhibited", "regulation", and "expression").

**Figure 3:**
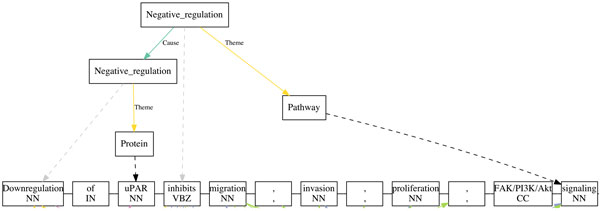
**Visualisation of a pathway event extracted by PWTEES**. Sentence: "Downregulation of uPAR inhibits migration, invasion, proliferation, FAK/PI3K/Akt signaling" (PMID: 21191179)).

In order to estimate the impact of removing some of the recognised gene names when they are part of pathway names, we compared the PWTEES results on the two test sets (P_TEST and PR_TEST) against the EVEX data. Example events that have been reported differently by EVEX and PWTEES are listed in Table 4. We note that some documents processed by PWTEES were not included in EVEX (denoted as 'N/A' in example #1 in Table 4). For other examples, we can see that introducing pathway entities does affect event extraction significantly. For instance, in example #3, EVEX only reports the occurrence of two genes (*CD40 *and *Fas)*, while PWTEES highlights that *CD40 *can inhibit *Fas*-mediated apoptosis. We can further see cases where the biological semantics is better represented, like in example #4. The emphasis of the sentence in example #4 is placed on the importance of the "*Ras/ERK1/2/ELK-1 *and *STAT3 *pathways". This is well captured by PWTEES. On the contrary, EVEX reports a sub-event of the up-regulation of *c-fos *promoter, thus missing the main finding expressed in the original sentence. In addition, if a gene name is embedded in a pathway name, TEES will only pick up the gene mention. This will result in a loss of information as a pathway mention refers to a different and more complex entity. Consider, for example, a pathway event extracted by PWTEES depicted in Figure 3. Here, TEES reports an event of "Downregulation of *uPAR*" and three gene names including *FAK, PI3K *and *Akt*. However, "*FAK/PI3K/Akt *signaling" is a single (pathway) name. PWTEES takes this correctly into account and reports the following event:

**Table 4:** EVEX and PWTEES differences on example sentences.

Example sentence	PMID	EVEX	PWTEES
Mutated BRAF, generates a constitutive activation of the mitogen-activated protein kinases (MAPK) signaling pathway	22863493	N/A	**T**: *Activation of MAPK pathway* **C**: *BRAF* **ET**: *Positive regulation*

PLD synergistically functions to activate the STAT3 signaling by interacting directly with the thyroid oncogenic kinase RET/PTC.	18498667	**T1**: *PLD* **T2**: *RET/PTC* **ET**: *Binding*	**T**: *PLD* **C**: *STAT3 signaling* **ET**: *Positive regulation*

CD40 stimulation inhibits cell growth and Fas-mediated apoptosis in a thyroid cancer cell line.	10223618	**Gene/Protein**: *CD40, Fas*	**T**: *Fas-medicated apoptosis* **C**: CD40 stimulation **ET**: *Negative regulation*

.. and that integration of the Ras/ERK1/2/ELK-1 and STAT3 pathways was required for up-regulation of the c-fos promoter by FMTC-RET	17209045	**T**: *c-fos promoter* **C**: *FMTC-RET* **ET**: *Positive regulation*	**T**: *up-regulation of the c-fos promoter by FMTC-RET* **C**: *Ras/ERK1/2/ELK-1 and STAT3 pathways* **ET**: *Positive regulation*

T - Theme, C - Cause, ET - Event Type

Theme: *FAK/PI3K/Akt *signaling

Cause: Downregulation of *uPAR*

Type: Negative regulation

### Application of PWTEES to the thyroid cancer corpus

With the parallel processing mentioned previously, we ran PWTEES on the whole thyroid cancer corpus. The number of unique interactions detected is listed in Table 5. To note, here pathway interactions only require either theme or cause to be a pathway. The table also lists the size of the EVEX-Human-TC-REL dataset we used for comparison. This dataset was retrieved from the EVEX human interaction dataset based on the thyroid cancer corpus (http://evexdb.org/download/network-format/Metazoa/Homo_sapiens.tar.gz).

**Table 5:** Unique interactions detected in the thyroid cancer corpus.

Type	Amount	Form
Genes/Proteins interactions	519	<Cause, Theme>

Binding pairs	145	<Theme1, Theme2>

Pathway interactions	313	<Cause, Theme>

EVEX-Human-TC-REL	599	<Source, Target>

To explore whether adding pathway information could provide a more comprehensive genetic space, we checked the genes involved in those interactions and compared those to the thyroid cancer related genes (TC-genes) extracted in our previous study [34]. A total number of 2,833 genes were included in TC-genes. Those genes were extracted via gene NER and normalization, and were shown to provide a more comprehensive coverage of genes related to thyroid cancer with respect that current manually curated datasets. For comparison, we also introduced other sets of genes:

· EVEX-genes: genes that participate in the EVEX interactions.

**Figure 4:**
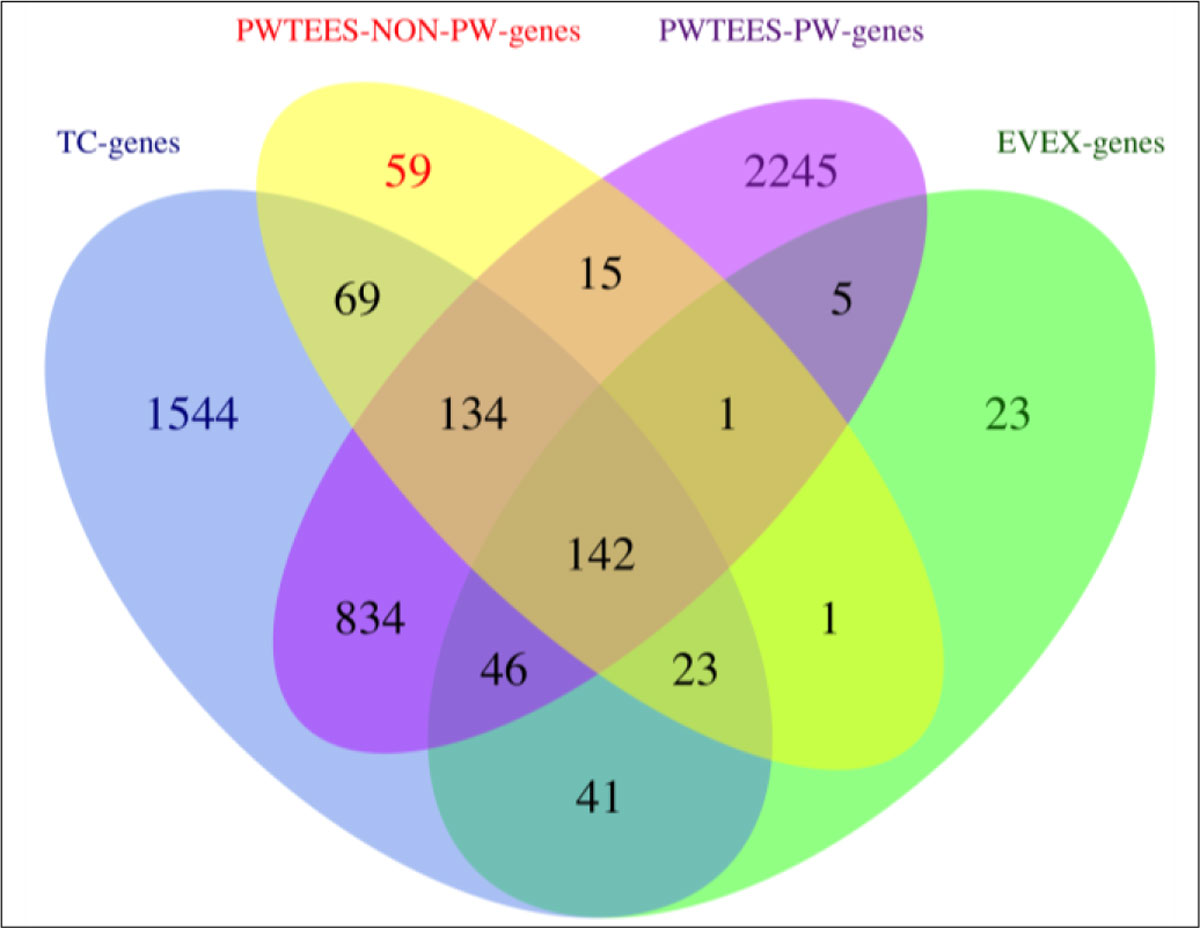
**Venn diagram for genes in different interaction sets**.

· PWTEES-NON-PW-genes: genes explicitly involved in non-pathway PWTEES interactions;

· PWTEES-PW-genes: genes involved in pathway PWTEES interactions and all genes in mentioned pathways;

· TC-genes: genes linked to thyroid cancer [34];

The result is depicted in Figure 4. The majority (83%) of the genes involved in non-pathway interactions are in the TC-genes dataset, that is, most genes in PWTEES-PW-genes are indeed linked to thyroid cancer. However, many genes in the TC-genes were not found in non-pathway interactions (87%), which reflects that without pathway details, many relevant genes would be missed. Finally, 40.8% of the TC-genes are involved in pathway events. The EVEX-genes cover only a small portion of TC-genes (282/2833, ~10 %). This highlights the importance of incorporating detailed pathway information when constructing a comprehensive molecular context.

### Molecular interaction networks

We constructed two networks as undirected graphs from the interactions mentioned above. The MERGE-PW network takes both genes and pathways as nodes, constructed as described in the Methods section. It contains 576 nodes and 3136 edges (see Additional file 1). The NON-PW network is based on gene/protein interactions and binding pairs (only containing genes). It contains 444 nodes and 628 edges (see Additional file 2).

We performed a topological analysis using the NetworkAnalyzer plugin in Cytoscape [39]. A number of commonly used network statistics are listed in Table 6. We can see that, in general, MERGE-PW is better connected than NON-PW, as the number of connected components for MERGE-PW is twice as small as that of NON-PW.

**Table 6:** Network statistics of NON-PW and MERGE-PW.

Network parameter	NON-PW	MERGE-PW
Clustering coefficient	0.117	0.150

Connected components	38	16

Network diameter	12	9

Characteristic path length	4.458	3.462

Average number of neighbours	2.710	7.170

Network density	0.006	0.012

Multi-edge node pairs	25	488

In MERGE-PW, the top 10 hubs are all pathways, as listed in Table 7. We also listed the top 10 gene nodes with the highest degrees of both networks in Table 8. For both the NON-PW and MERGE-PW networks, all top-10 hub genes are highly ranked in the TC-genes by document-level frequency. However, we can observe that some genes are more connected in the MERGE-PW network than in NON-PW. For instance, *HRAS *(ID: 3265), one of the most common mutated genes for thyroid cancer [40], does not have many neighbors in NON-PW (with a degree of 10), but it has the sixth highest degree among all gene nodes in MERGE-PW. Similarly, *MYC *(ID: 4609) was highly ranked in the TC-genes but it has only one neighbor in NON-PW. This highlights the importance of integrated pathway data.

**Table 7:** Top 10 hubs in the MERGE-PW network.

**No**.	Hub (pathway name)	Degree
1	JAK/STAT3 pathway	276

2	MAPK/ERK pathway	245

3	TSHR signaling	159

4	PI3K/Akt pathway	141

5	Apoptosis	111

6	TSHR-induced G(q) signal transduction	92

7	EGFR signaling	83

8	TGFbeta transduction	81

9	epidermal growth factor receptor 1 signaling	78

10	T3/TR signaling	70

**Table 8:** Top 10 gene nodes with the highest connectivity.

**No**.	NON-PW			MERGE-PW genes		
	**Hub (Gene ID)**	**Symbol**	**Degree**	**Hub (Gene ID)**	**Symbol**	**Degree**

1	5979	*RET*	58	207	*AKT1*	72

2	7157	*TP53*	24	5979	*RET*	63

3	207	*AKT1*	23	5594	*MAPK1*	57

4	7422	*VEGFA*	21	3265	*HRAS*	49

5	1950	*EGF*	19	4609	*MYC*	46

6	595	*CCND1*	18	5595	*MAPK3*	46

7	5594	*MAPK1*	15	6774	*STAT3*	45

8	673	*BRAF*	14	7157	*TP53*	45

9	6774	*STAT3*	14	1950	*EGF*	43

10	5727	*PTCH1*	14	595	*CCND1*	42

We also analysed bottlenecks using the cyto-Hubba plugin for Cytoscape [41]. Bottlenecks are nodes with high betweeness centrality, which is measured by the number of shortest paths passing through a node [42]. Table 9 shows the top 10 bottlenecks in the MERGE-PW network. These include apoptosis, which is important in all types of cancer. Another pathway bottleneck is "Epidermal growth factor receptor 1 signalling", which has been identified as a therapeutic target for cancer [43]. Two gene bottlenecks that are not in the top 10 hubs are *PIK3R2 *(ID: 5296) and *TNF-alpha *(ID: 7124). *PIK3R2 *is a regulatory component of PI3K, a major member of the PI3K/Akt pathway, one of the top hubs in the MERGE-PW network (see Table 9). *TNF-alpha *encodes a multifunctional cytokine that has been implicated in many diseases including cancer [44].

**Table 9:** Top 10 bottlenecks in the MERGE-PW network.

Rank	Name	Betweeness Centrality
1	Apoptosis	110.0

2	5296 (*PIK3R2*)	45.0

3	5979 (*RET*)	44.0

4	Epidermal growth factor receptor 1 signaling	31.0

5	7157 (*TP53*)	27.0

6	207 (*AKT1*)	20.0

7	Cell cycle	20.0

8	1950 (*EGF*)	18.0

9	7124 (*TNF-alpha*)	18.0

10	5594 (*MAPK1*)	17.0

## Conclusions

In this paper we presented an approach to enrich the molecular context of diseases by applying large-scale text mining of events involving genes and pathways. We extended a state-of-the-art text mining system by introducing pathway NER to identify interactions involving both genes/proteins and pathways. We then applied the expanded system on a corpus of thyroid cancer and generated interactions involving both genes and pathways. We were able to demonstrate that integrating information about pathways can provide additional molecular insights, highlighting a few key genes and pathways. To facilitate further exploration of thyroid cancer carcinogenesis, the whole MERGE-PW network is given in Additional file 1.

The PWTEES system is however not specific to thyroid cancer: it can be applied to studies of other complex diseases. In future work, we aim to run PWTEES on the whole MEDLINE and PMC Open Access set to generate a large scale dataset that can provide a searchable disease-sensitive interface for interaction events involving pathways.

## Availability

The code is available at: https://github.com/chengkun-wu/PWTEES

## List of abbreviations used

NER - named entity recognition; TEES - Turku Event Extraction System; TC - thyroid cancer; PWTEES - Pathway TEES.

## Competing interests

The authors declare that they have no competing interests.

## Authors' contributions

CW designed and developed the system for the detection of gene/pathway events, and drafted the manuscript. GN provided support and guidance from the text mining perspective and JMS from the systems biology perspective. SP provided expertise on high performance computing. GB provided expertise for the thyroid cancer case study; GN and JMS conceived and supervised the project. All authors read and approved the final manuscript.

## Supplementary Material

Additional File 1 **The MERGE-PW network in SIF format**. Click here for file

Additional File 2 The Cytoscape project file (binary data, viewable by Cytoscape) containing both the MERGE-PW network and the NON-PW network in *.zip format. Click here for file

## Declarations

The publications charges for this article were funded by an RCUK block grant to the University of Manchester.

This article has been published as part of *BMC Systems Biology *Volume 9 Supplement 6, 2015: Joint 26th Genome Informatics Workshop and 14th International Conference on Bioinformatics: Systems biology. The full contents of the supplement are available online at http://www.biomedcentral.com/bmcsystbiol/supplements/9/S6.
